# A Review of Biofilm Formation of *Staphylococcus aureus* and Its Regulation Mechanism

**DOI:** 10.3390/antibiotics12010012

**Published:** 2022-12-22

**Authors:** Qi Peng, Xiaohua Tang, Wanyang Dong, Ning Sun, Wenchang Yuan

**Affiliations:** 1 Guangzhou Key Laboratory for Clinical Rapid Diagnosis and Early Warning of Infectious Diseases, KingMed School of Laboratory Medicine, Guangzhou Medical University, Guangzhou 510180, China; 2 Guangzhou First People’s Hospital, School of Medicine, South China University of Technology, Guangzhou 510180, China

**Keywords:** *Staphylococcus aureus*, biofilms, extracellular matrix, quorum sensing, antibiofilm, antibiotic resistance

## Abstract

Bacteria can form biofilms in natural and clinical environments on both biotic and abiotic surfaces. The bacterial aggregates embedded in biofilms are formed by their own produced extracellular matrix. *Staphylococcus aureus* (*S. aureus*) is one of the most common pathogens of biofilm infections. The formation of biofilm can protect bacteria from being attacked by the host immune system and antibiotics and thus bacteria can be persistent against external challenges. Therefore, clinical treatments for biofilm infections are currently encountering difficulty. To address this critical challenge, a new and effective treatment method needs to be developed. A comprehensive understanding of bacterial biofilm formation and regulation mechanisms may provide meaningful insights against antibiotic resistance due to bacterial biofilms. In this review, we discuss an overview of *S. aureus* biofilms including the formation process, structural and functional properties of biofilm matrix, and the mechanism regulating biofilm formation.

## 1. Introduction

Biofilm is an organized bacterial population and refers to the membrane-like extracellular matrix (ECM) formed by the adhesion of bacterial colonies and extracellular polymeric substances (EPS) such as polysaccharides, nucleic acids, and proteins secreted by bacteria during the growth process [1]. The interaction between EPS and bacterial aggregates endows biofilm with cohesion and viscoelasticity [2]. As a result, bacteria can attach to both biotic and abiotic surfaces. The formation of pathogenic biofilm plays an important role in causing chronic persistent infection [3]. Currently, researchers generally believe that more than 80% of chronic infections are mediated by bacterial biofilms [4]. *Staphylococcus aureus* (*S. aureus*) is prevalent in hospital environments. It attaches to and persists on host tissues and indwelling medical devices. This may cause skin and soft tissue infection, osteomyelitis, endocarditis, pneumonia, bacteremia, etc. [5,6,7,8]. These infections are difficult to cure due to the biofilm formed that enhances the resistance of *S. aureus* to antibiotics [9]. Additionally, biofilm formation is considered to be a protected growth mode for bacteria to adapt to harsh environments [10]. The biofilm acts as a barrier to create a stable internal environment for bacterial cell activity and protects bacterial cells from adverse conditions including extreme temperature, nutritional restriction and dehydration, and even antibacterial drugs [11]. Consequently, bacteria can settle quickly and protect themselves from host defense mechanisms and then promote long-term infection by enhancing adhesion to the host surface. Biofilm is therefore the first self-protection line of bacteria. It has been known that biofilm-forming bacteria are resistant to most antibiotics [12]. Most clinical antibiotics are developed targeting planktonic microbial cells. Antibiotics targeting planktonic cells may exert selective pressure on microorganisms, thus giving them a survival advantage over susceptible competitors [13]. Therefore, antibiotic therapy against biofilm usually requires long-term use antibiotics at high doses [14]. However, chronic treatment with such antibiotics may lead to increase the risk of antibiotic resistance and drug toxicity [15]. Due to the highly complex and rapid adaptability of biofilm population [16], an in-depth understanding of biofilm formation mechanism may provide new insights for the development of effective infection control strategy against biofilms [17,18].

## 2. Biofilm Formation Process

**Figure 1:**
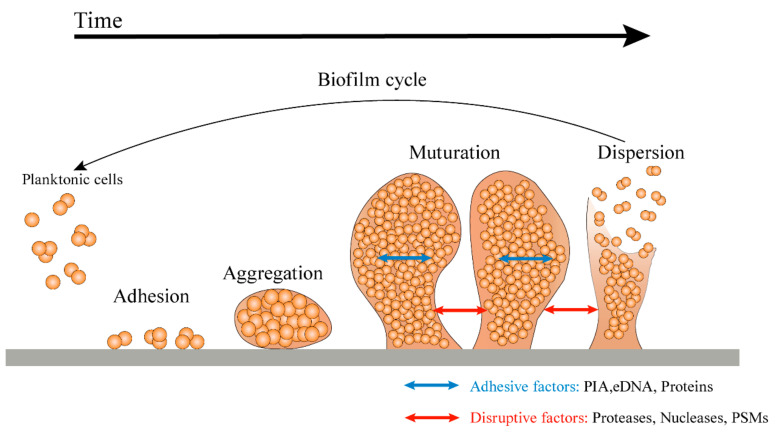
A model of the growth cycle of biofilm. In the first step of biofilm formation, planktonic cells attach to the surface via surface-associated proteins. After attachment, the cells gradually aggregate and begin to produce ECM, thus forming microcolonies. With cell division, a mature biofilm is gradually formed. Finally, in the separation stage, enzymes such as protease, nuclease, and a quorum sensing system promote the dispersion of biofilm, allowing the bacterial cells to detach from the biofilm and return to a planktonic state to colonize new ecological niches [40].

The formation of three-dimensional biofilm by bacteria is a complex process. It is generally divided into four stages: adhesion, aggregation, maturation, and dispersion (Figure 1) [19].

During the adhesion stage, *S. aureus* planktonic cells use a range of different factors and relevant regulatory mechanisms, such as the expression of cell wall-anchored protein (CWP), adhesin, and eDNA, to promote the combination with the host [20]. The most characteristic of these regulations is the organization of microbial surface components recognizing adhesive matrix molecules (MSCRAMMs) that mainly include fibronectin-binding proteins (FnBPs, including FnbA and FnbB) [21], fibrinogen-binding protein (Fib), clumping factors (ClfA, ClfB) [22], and serine-aspartate repeat family proteins (SdrC, SdrD, and SdrE) [23]. These components mediate bacterial adhesion to natural tissues and biomaterial surfaces. In addition, bacterial appendages, such as flagella, cilia, and pili, allow for more permanent adherence of bacteria to surfaces [24].

After adhering to surfaces, the adherent bacterial cells begin to divide and accumulate in the presence of a sufficient nutrient source [19]. During the aggregation stage, bacteria regulate biofilm formation by sensing environmental signals that trigger regulatory networks and intracellular signal molecules. Then, bacteria continue to proliferate and thicken to form a biofilm [25]. The biofilm formed can provide resistance against the human immune system and antibiotics [26]. Bacterial cells proliferating in the matrix may lose direct contact with the graft surface and host protein and mainly depend on cell–cell and cell–EPS adhesion [27].

During the maturation stage, the structure of biofilm is highly structured and a compact three-dimensional mushroom or tower structure is formed [28]. A large number of pipes around the microcolony is constructed to promote the transport of nutrients to the deep layer of the biofilm [29]. Mature biofilms have diverse and unique metabolic structures that enable them to resist harmful environmental factors and stress drivers [30].

EPS can be attached by many single bacterial cells to form microcolonies, which are the basic units of biofilm structures [31]. Once microcolonies are formed, the bacterial biofilm continues to thicken and disperse the biofilm under the influence of specific genetic regulations or external factors. The process of dispersion may involve multiple steps, including the production of exoenzymes and surfactants to degrade EPS [32] and physiological changes that prepare cells for conditions outside the biofilm [33]. The dispersed bacteria form planktonic bacteria, which in turn can colonize other sites and form biofilm under certain conditions, thus forming a cyclic process. 

The dispersion step is the final stage of the biofilm life cycle and the beginning of another life cycle [34]. During the growth and development of biofilm, surfactant phenol-soluble modulins (PSMs) are a key effector molecule for the dispersion and transmission of *S. aureus* biofilms [35]. PSMs are characterized by amphiphilicity α-helical secondary structure, which gives them surfactant-like properties [36]. These properties can destroy the non-covalent force in the biofilm matrix, forming the necessary channels to transport nutrients to the deeper layer of the biofilm. It also provides the necessary destructive force to spread the biofilm masses to the distal position [37]. PSMs of *S. aureus* not only exist in soluble form, but also aggregate into amyloid fibers to eliminate the biofilm-degrading activity of monomeric PSMs peptides and stabilize the biofilm structure [38]. In *S. aureus*, α-PSM1 and α-PSM4 peptides are the main amyloid proteins involved in the α-PSMs fibril production [39].

## 3. Biofilm Formation Mechanism

### 3.1. Polysaccharide Intercellular Adhesion(PIA)-Dependent Mechanism

**Figure 2:**
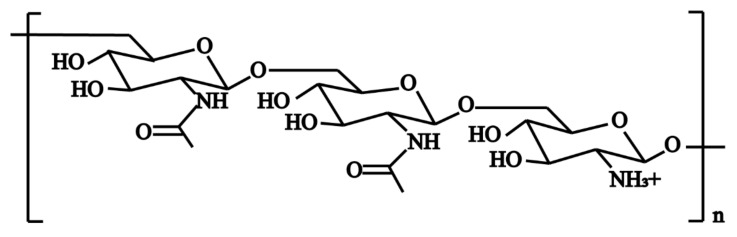
The structure of polysaccharide intercellular adhesin (PIA). PIA is a beta-linear homoglycan composed of 1,6-linked N-acetylglucosamine residues, 15–20% of which are deacetylated and therefore positively charged [41].

**Figure 3:**
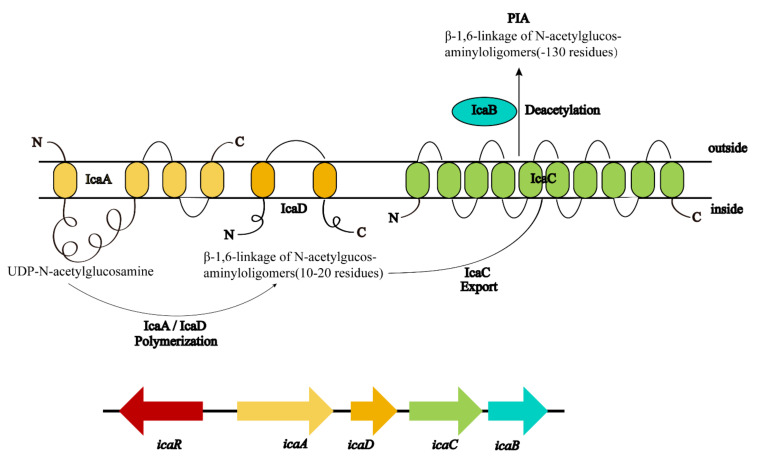
Genetic encoding and biosynthesis of PIA. PIA is synthesized from the product of *icaADBC* operon. *IcaA* and *IcaD* are two membrane proteins that polymerize N-acetylglucosamine from the activated precursor UDP-N-acetylglucosamine monomers. This chain may be exported by the membrane protein *IcaC*. *IcaB* is an acetylase attached to the outer surface of bacteria. By deacetylation of residues, *IcaB* introduces a positive charge into the originally neutral PIA molecule [41].

Among the polymeric molecules involved in ECM, polysaccharide intercellular adhesion (PIA) (also known as poly-N-acetylglucosamine; PNAG) is an important factor in *S. aureus* biofilm formation [41]. PIA has a cationic property (Figure 2) and plays an important role in the adhesion and aggregation stage [42]. In mutual strains lacking PIA, the ability of bacterial cells adhering to each other is decreased significantly. In *S. aureus*, the mechanism of biofilm formation is controlled by the production of PIA through proteins encoded by *icaADBC* operon in the *ica* locus (Figure 3) [43]. In this mechanism, *icaA* and *icaD* genes are essential in the regulation of biofilm formation. The product of the *icaA* gene is N-acetylamino-glucosamine transferase which is a transmembrane protein [44]. The product of the *icaD* gene is the chaperone protein of *icaA*. It maintains the correct folding of *icaA* and increases the specificity of *icaA* to polymers [45]. The product of the *icaC* gene is a transmembrane protein that mediates the transfer of the newly synthesized PIA to the cell surface [46]. The product of the *icaB* gene is a deacetylase responsible for the deacetylation of mature PIA. This deacetylation gives the polymer a net positive charge, which is required for adhesion onto the cell surface and intercellular adhesion [47]. The maximum length of poly N-acetylglucosamine oligomer produced by *icaAD* is 20 residues. Longer oligomer chains are synthesized only when *icaAD* is co-expressed with *icaC*. PIA also increases biofilm retention and its resistance to antimicrobial peptides (AMPs) through deacetylation [48]. Non-deacetylated polyglucosamine in the homogenous *icaB* mutant cannot adhere to the surface of bacterial cells or mediate the biofilm formation [49]. The *icaADBC-*mediated polysaccharide production is an important mechanism for biofilm formation and contributes to the early growth of bacteria. Additionally, it is believed that the *ica* operon is under phase variation, which has a role in slipped strand mispairing and leads to an on/off switch for expressing the products [50].

### 3.2. Protein-Dependent Mechanisms

Some studies reveal that the strains without *ica* operon can also form biofilm [51,52]. Among the strains forming biofilm, the mutation of the *ica* gene does not affect the formation of biofilm. When treated with proteases, the biofilm of these strains can be depolymerized [51]. This indicates that there are proteins involved in biofilm formation through other mechanisms independent of the *ica* operon. 

The *bap* gene of *S. aureus* encodes a surface protein Bap (biofilm-associated protein) containing 2276 amino acids. Bap was identified as the main determinant of successful surface adhesion and intercellular adhesion during biofilm formation. It promotes the initial adhesion of bacteria to biological and abiotic surfaces and intercellular adhesion through an extracellular polysaccharides-independent mechanism [52]. All *S. aureus* strains carrying the *bap* gene have high adhesion and strong biofilm-forming ability, indicating that there is a strong correlation between the existence of this protein and the biofilm-forming ability onto an abiotic surface. The N-terminal region of Bap is released into ECM and assembled into amyloid fibers to help the construction of *S. aureus* biofilms [53]. The Bap core domain contains C repeats, which are predicted to fold into new structures and participate in cell adhesion. The Bap C-terminal contains a typical cell wall attachment region [54]. During infection, Bap promotes persistence in the mammary gland by enhancing adhesion to epithelial cells and binds to host receptors to prevent cellular internalization, thereby interfering with the FnBPs-mediated invasion pathway [53].

Fibronectin-binding proteins are multi-structural domain glycoproteins (440 kDa), which are found in almost all tissues and organs and biological fluids and play an important role in cell adhesion and migration [55]. The N-terminal A structure of fibronectin-binding proteins A (FnbA) is structurally and functionally related to cohesion factor ClfA and *Staphylococcus epidermidis* SdrG protein. FnbA binds to fibrinogen at the N2 and N3 ends of the A structural domain through changes in the DLL (dock, lock, and latch) mechanism to form a highly stable complex [56], promotes the accumulation phase and initial adhesion phase of the biofilm, and increases bacterial aggregation [27].

The attachment of clumping factors promotes colonization of *S. aureus* in the host, facilitates biofilm formation, and causes virulence by binding soluble fibrinogen for immune escape [57]. However, even in the absence of fibrinogen, the biofilm of some strains is dependent on increased ClfB activity in the absence of calcium. ClfB accumulates on the bacterial surface and mediates biofilm formation [58].

*S. aureus* surface protein G (SasG) causes intercellular adhesion and promotes biofilm formation through zinc-dependent dimerization [59]. The fibrillar nature of SasG can mask the binding of *S. aureus* MSCRAMM to their ligands and also promote biofilm formation [60]. *S. aureus* surface protein C (SasC) mediates cell cluster formation, intercellular adhesion, and biofilm formation, but SasC does not mediate the interaction with fibrinogen or fibronectin [61].

The carboxyl terminus of serine–aspartate repeat family proteins contains motifs required for cell-wall anchoring. SdrC mediates strong cellular interactions with hydrophobic surfaces, which may be related to the initial attachment of biological materials, the first stage of biofilm formation [62], while SdrC binds with low-affinity homophilic bonds and promotes cell adhesion as well as biofilm formation [62]. SdrD is an important key factor in the ability of *S. aureus* to survive and evade the blood’s intrinsic immune system. SdrD promotes *S. aureus* adhesion to exfoliated nasal epithelial cells [63] and human keratin-forming cells in vitro [64]. It also promotes abscess formation in vivo [65]. SdrE traps the C-terminal tail of complement factor H (CFH) by a unique mechanism and isolates CFH on the surface of *S. aureus* to evade complement [66].

The collagen-binding adhesin (CAN) was originally reported to be necessary and sufficient for the binding of *S. aureus* to collagen-rich stromal cartilage [67]. CAN is a virulence factor in several animal models of infectious diseases. It also functions as an adhesin [68,69]. CAN is also a potential complement inhibitor that disrupts the molecular mechanism of complement activation and represents a potential immune evasion strategy that is associated with the development of multiple diseases [70].

### 3.3. Extracellular DNA (eDNA)-Dependent Mechanism 

The mature *S. aureus* biofilm is sensitive to the external addition of DNase I, indicating that eDNA is a structural component of the biofilm matrix [71]. Due to the negative charge of DNA polymer, eDNA may participate in the early adhesion stage and mature stage of biofilms as an electrostatic polymer and play a basic structural function in the structural integrity of biofilms [72]. Its mechanism is to connect PIA and biofilm-related proteins and other biofilm components to stabilize the biofilm structure [73,74]. At the same time, eDNA also introduces favorable acid-base interactions to enhance adhesion and surface aggregation [75]. The accumulation of eDNA in the biofilm and infection site can acidify the local environment and promote antibiotic resistance phenotype [76]. It was found that eDNA also mediated horizontal gene transfer though conjugation of plasmids between cells in biofilms [77] and neutralized the important effector molecules of innate immunity such as AMPs by binding and isolating cations from the surrounding environment [78]. In *Staphylococcus epidermidis*, eDNA has also been found to be thermodynamically favorable interacting with positively charged vancomycin. It can reduce the potency of vancomycin, inhibit the transport of vancomycin in the biofilm, and thus protect the bacteria embedded in the biofilms [79]. eDNA is released through cell death and lysis and it is mainly regulated by the *cidA* gene. *cidA* encodes a responser of cell wall hydrolase activity and regulates cell death. *cidA* mediated cell lysis contributes to *S. aureus* biofilm formation in vivo and in vitro [80]. The inhibition of *cidA* activity by *lgrAB* operon can inhibit cell lysis and adhesion in the process of biofilm formation. Mutation of *lgrAB* operon leads to the formation of more adherent biofilms and higher eDNA contents [81]. Although these are the main components of the biofilm matrix, the exact composition of the biofilm matrix may vary and depends on matrix availability and physical factors.

## 4. Regulation Mechanism of Biofilm Formation

Biofilm formation is a social group behavior. Each of the steps from initial attachment to the dispersion and transmission of mature biofilm is strictly controlled by multiple regulatory systems or regulators [82]. 

### 4.1. Regulation of Quorum-Sensing System-Mediated Biofilm Formation

Intercellular signal transduction, commonly known as quorum sensing, is an internal communication system of bacteria. Bacteria detect the changes in the number of individual bacterial cells or other bacterial populations in the surrounding environment based on the changes in the concentration of a specific signal autoinducer. When the signal molecule reaches a certain threshold, the expression of relevant genes in bacteria is initiated to adapt to the changes in the environment [83]. The quorum sensing system usually involves signal transduction pathways that regulate biofilm formation, virulence, binding, antibiotic resistance, motility, and sporulation [84]. The quorum sensing system of *S. aureus* includes an accessory regulatory factor (Agr) system and LuxS/AI-2 system [85]. These two systems reduce biofilm formation in two different ways. The Agr system dissociates bacterial biofilm by upregulating the transcription of RNAIII, while the LuxS/AI-2 system reduces the expression of PIA.

**Figure 4:**
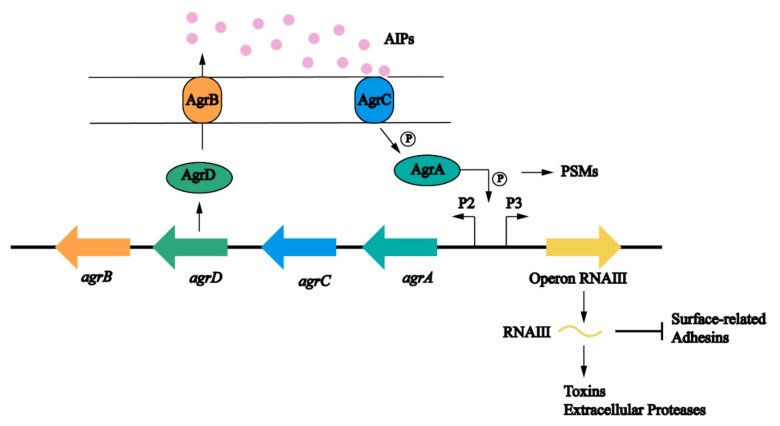
The role of the Agr quorum sensing system in biofilm formation in *S. aureus*. The Agr system is controlled by *agr* operon. AgrD is the precursor of self-induced peptides (AIPS), which is modified by AgrB and secreted into the extracellular matrix. AIPs are intracellular signal molecules that respond to cell density. When the bacterial density increases, AIP activates the transmembrane protein AgrC. Phosphorylated AgrC further activates AgrA and finally promotes the expression of target genes. AgrA acts on P2, which regulates the Agr protein, and P3, which can activate RNAIII expression. RNAIII is the effector molecule of *agr* locus. RNAIII induces the expression of virulence factors, such as protease and toxin. On the other hand, RNAIII also inhibits the expression of surface adhesion proteins [83].

**Figure 5:**
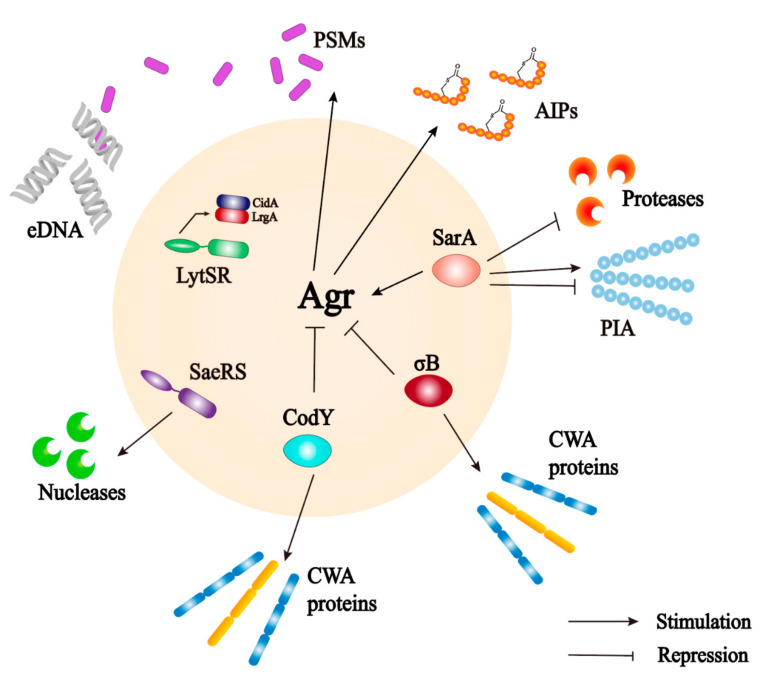
The interaction between Agr quorum sensing system and some important biofilms regulators (LytSR TCS, SigB, CodY and SarA) [20].

The *agr* locus is a complex polygenic system (Figure 4). It responds to bacterial cell density and controls the expression of *S. aureus* adhesion and extracellular protein. The regulation of the Agr system on biofilm is multifaceted and is mainly involved in the adhesion, maturation, and dispersion stages [40]. The Agr system uses an autoinducing peptide (AIP) as the signal molecule of cell density [86]. The *agr* locus encodes a two-component quorum sensing system consisting of two relatively independent transcription units driven by P2 and P3 promoters [87]. The P2 promoter starts the transcription of RNAII. The RNAII transcript harbors four open reading frames including *agrA*, *agrB*, *agrC,* and *agrD*, which encode the proteins required for AIP biosynthesis, transport, signal sensing, and regulation of target genes [88]. AgrC is a signal transduction factor with sensor histidine protein kinase activity; AgrA is a response regulator; AgrD is a precursor of the AIP; and AgrB is a multifunctional endopeptidase and chaperone protein. On the one hand, AgrB participates in the processing of AgrD as a protease to make it a mature AIP, but on the other hand, it acts as an oligopeptide transporter that helps secrete mature AIP out of cells [89]. The P3 promoter initiates the transcription of RNAIII. When the concentration of AIP in the environment reaches a certain threshold, it binds to and activates the histidine kinase AgrC, which leads to autophosphorylation and initials the signal transduction process [90]. AgrC phosphorylates AgrA after activation, which in turn induces the P3 promoter to transcriptionally express RNAIII; the main effector molecule of quorum sensing system [88]. RNAIII positively regulates the expression of toxin genes, prevents the translation of the repressor of toxin (Rot) [91], and reduces the expression of several surface adhesins, which are negatively associated with biofilm formation [92]. At the same time, an increased level of the AIP can promote the depolymerization of *S. aureus* biofilms by increasing the secretion of extracellular protease [85,93]. Different from other targets, the production of PSMs is generated by the direct binding of AgrA to its promoter [94,95]. Upregulation of *agr* leads to an increase in PSMs and promotes maturation and spreading of biofilm. In addition, some nutrients can affect the biofilm formation through the Agr system. Studies have shown that glucose strongly inhibits the expression of the P3 promoter. In established biofilms, glucose consumption activates the Agr system and leads to biofilm diffusion [93]. In *S. aureus*, some regulatory systems are interconnected with the Agr system that regulates the response to changes in environmental conditions and the development of biofilms (Figure 5) [20].

LuxS/AI-2 quorum sensing system is shared by both Gram-negative and Gram-positive bacteria. It is mediated by the signal molecule AI-2 (furanyl borate diester) synthesized by *luxS* gene (AI-2 synthase). It enables bacteria to make collective decisions about the expression of a specific set of genes. The precursor of AI-2 is 4,5-dihydroxy-2,3-pentanedione (DPD) [96]. LuxS/AI-2 quorum sensing system that exists in *S. aureus* plays a role in the regulation of biofilm formation [97]. Two early studies have shown that LuxS is a negative regulator of biofilm formation. Biofilm formation is significantly increased in *luxS* mutant strains compared with wild strains. A study reported that the transcript level of *icaR* was significantly reduced in *luxS* mutants, while *icaA* expression was significantly increased, suggesting that AI-2 represses *icaADBC* transcription through activation of *icaR* [98]. Moreover, when a low nanomolar concentration range of DPD was added, the biofilm formation was changed. On the contrary, when higher concentrations of DPD were added, no effect on biofilm formation was observed [98]. Another study found that Rbf was a positive regulator of biofilm. It promotes PIA-dependent biofilm formation in *S. aureus* by binding to the *sarX* promoter, upregulating *sarX* transcription, and indirectly downregulating *icaR* expression [99]. The transcription level of *rbf* was increased in *luxS* mutant strains. The transcription of the *rbf* gene could also be restored to normal when supplemented with *luxS*-containing plasmids or effective concentrations of exogenous AI-2. The results suggest that *luxS* may suppress *rbf* expression and reduce the transcription level of *icaA* through AI-2-mediated signaling, thereby reducing PIA-dependent biofilm formation [100]. In contrast to the above study, another study observed upregulation of the *icaADBC* by AI-2 in *S. epidermidis*. The expression of the *icaADBC* was also upregulated when micromolar concentrations of DPD were added [101]. This result suggests that the effect of signal molecule AI-2 may be very different for each species, even depending on the concentration.

### 4.2. The Global Response Regulator

Staphylococcal accessory regulator (SarA) is a DNA-binding protein encoded by *sarA* locus [102]. It is the main global regulator of many virulence determinants and directly regulates the expression of some virulence factors [103]. *sarA* locus is necessary for *ica* operon transcription, PIA/PNAG production, and biofilm formation of *S. aureus* [104]. SarA regulates biofilm formation through the *agr*-dependent pathway, binds to the *agr* promoter to stimulate transcription of RNAIII, and cascades and regulates downstream target genes [105,106]. SarA can control gene expression by directly interacting with target gene promoters through the *agr*-independent pathway [104]. In addition, SarA activates the P2 promoter and promotes transcription by bending the DNA region of the *agr* promoter, thereby enhancing *agr* dimer interactions to upregulate *agr* expression [107]. A sequence with 58% homology to the predicted recognition sequence of *sarA* exists at 70 nt upstream of *ica* start codon in *S. aureus*. The purified SarA binds to this sequence with high affinity to upregulate *ica* operon expression and promote biofilm formation [108]. SarA not only induces the transcription of *ica* operon but also of its suppressor *icaR*, indicating that SarA may prevent the excessive production of PIA [109]. Another role of SarA in biofilm formation is the inhibition of extracellular proteases production [110]. *sarA* mutants have a global impact on the abundance of many *S. aureus* transcripts, producing high levels of proteases, such as metalloproteinase Aur, serine protease SspA, cysteine protease SspB and ScpA, resulting in the inability to form biofilms [110]. Only by simultaneously eliminating the production of these extracellular proteases, the biofilm formation and virulence of *sarA* mutants can be fully restored [111]. In summary, SarA is an important regulator controlling the biofilm formation of *S. aureus* through a variety of mechanisms.

σ^B^ is a product of *sigB* operon and is the main regulator of *S. aureus* response to environmental stress. It plays an important role in the production of bacterial drug resistance, biofilm formation, and the expression regulation of virulence-related genes [112]. Under osmotic stress, the biofilm formation of *S. aureus* wild strain MA12 is significantly stimulated, but the *sigB* deletion mutation eliminates the biofilm formation. After supplying the *sigB*-containing plasmid, biofilm formation could be restored to normal under conventional conditions or stimulated by osmotic stress [113]. However, another study reported that the *sigB* deletion mutant still formed biofilm effectively, suggesting that *sigB* could regulate *S. aureus* biofilm in a strain-dependent manner [114]. σB also mediates an increase in SarA level and decreases the level of RNA III of the Agr system, leading to growth-stage-dependent differences in some virulence factors [115]. Moreover, it mediates the production of several cell surface proteins related to the early adhesion of biofilms, such as FnbA and ClfA. σ^B^ promotes the transcription of *fnbA* in early growth and significantly upregulates the transcription of *clfA* in late growth [116]. However, various exoprotein genes that are important for biofilm dispersal are repressed by σ^B^ [117].

In *S. aureus*, the global transcription factor CodY acts mainly as a repressor of metabolic and virulence genes, directly or indirectly regulating more than 200 genes [118]. Under adequate nutritional conditions, CodY interacts with its effector molecules, leading to conformational changes in CodY that enhance the affinity of CodY to its DNA binding sites [119]. CodY strongly inhibits the *agr* locus [120]. Some studies have shown that CodY may bind to a locus in *agrC* in vitro [121,122], but this binding does not affect the in vivo transcription of *agrA*, suggesting that CodY does not control *agr* gene expression through direct binding within the *agr* locus [122]. The identification of CodY regulatory Agr system targets reveals undoubtedly a new regulatory pathway affecting most virulence genes in *S. aureus*. CodY regulates biofilm formation by mediating *ica* expression and PIA production [117,123]. In the *codY* deletion mutant, the *icaA* was overexpressed; in the *icaR* deletion mutant, the transcript level of *icaA* gene was extremely low and the amount of biofilm formation was low; in the *icaR* and *codY* double mutant, the transcript level of *icaA* was similar to that of the *codY* mutant, but the amount of biofilm formation was three times more than that of the *codY* mutant, indicating that *codY* may override the *icaR*-mediated loss of the *ica* locus repression and that *codY* is epistatic to *icaR* [118]. In addition to regulating PIA-dependent biofilm, CodY also represses the exoprotease and thermonuclease (*nuc*), which are important regulators of biofilm formation [118,124]. *nuc* is directly controlled by the SaeRS system, and in *codY* null mutants, *nuc* overexpression requires SaeR, indicating that the *codY* overexpression phenotype is at least partially via the SaeRS system [118]. In addition, CodY regulates the *sae* locus in a Rot-dependent and Rot-independent manner [125].

### 4.3. ica Operon

The enzyme required for PIA synthesis is encoded by *icaADBC. icaR* are located upstream of *icaADBC* and belong to the TetR family encoding transcriptional repressors of the *ica* locus [126]. IcaR is a DNA-binding protein that negatively regulates *icaADBC* expression by binding to the upstream region of the *icaA* start codon [127]. In addition to IcaR, a second *icaADBC* direct repressor has been identified. This regulator, named TcaR (teicoplanin-associated locus regulator), belongs to the MarR family of transcriptional regulators [128]. The putative binding sequence of TcaR has been identified in the promoter region of the *icaADBC* operon and has been shown to function as a direct repressor by TcaR [129].

Existing studies have pointed out that *S. aureus* isolates carrying the deletion of the 5 bp motif “TATTT” between the *icaR* and *icaA* intergenic region exhibit a mucoid phenotype, resulting in the increased transcription of *ica* and inducing excessive production of PIA/PNAG in *S. aureus* [130]. Through the electrophoretic mobility shift assay and DNaseI footprint assay of recombinant IcaR, it was found that the recombinant IcaR protected a 42 bp region upstream of the *icaA* gene, but not with the region containing 5 bp motif. It shows that the transcriptional control of 5 bp at *ica* site is independent of *icaR* [131]. The effect of the 5 bp motif on *icaADBC* expression is considered to be controlled by other undetermined repressors. Whole-genome sequencing and microarray analysis revealed that another clinical isolate with a mucus phenotype contained a hypothetical transcriptional regulatory gene with a spontaneous mutation that was expressed at a significantly higher level than in a control strain without biofilm formation [132]. This gene was named “regulator of biofilm” (*rob*). Rob is a DNA-binding protein that shares homology with the TetR family. Rob was experimentally shown to recognize and bind a 25 bp region between the *icaR* and *icaA* intergenic regions (including the 5 bp motif). In the absence of 5 bp, Rob cannot bind to this region, resulting in excessive biofilm formation and is a novel repressor of the *ica* locus [132]. Rob was first discovered as the *gbaAB* (glucose-induced biofilm access gene) operon. GbaAB can participate in the regulation of the multicellular aggregation step of glucose responsive *S. aureus* biofilm formation through *ica* locus and PIA [133]. However, Rob affects biofilm formation in a glucose-independent manner [132]. Although the strains used in these two studies are different, the potential mechanisms need to be further explored.

### 4.4. Two-Component Regulatory System

The SrrAB TCS (*Staphylococcal* respiratory response regulator) is a major regulator of respiratory growth and virulence in *S. aureus*, which is critical for survival under environment conditions such as hypoxic and oxidative [134]. The oxygen utilization plays an important role in *S. aureus* virulence by regulating toxin production and biofilm formation [135]. SrrAB TCS sensor kinase SrrB responds to oxygen in the environment by autophosphorylation and the effector molecule SrrA activates or represses the regulation of target genes [136]. Purified phosphorylated SrrA binds to a 100 bp DNA sequence upstream of *icaA* in a concentration-dependent manner to induce transcription of *icaA* and PIA production under anaerobic conditions [137]. In particular, the introduction of insertional mutations in *srrA* resulted in increased PIA production but reduced biofilm formation [138]. Meanwhile, the cysteine residues within the SrrB Cache domain form redox-sensitive disulfide bonds, which are required for biofilm formation and virulence expression under anaerobic and microaerobic conditions [134]. Another study showed that SrrAB-dependent biofilms increased with decreased respiratory activity due to fermenting cells increasing eDNA and proteins in an AtlA murein hydrolase-dependent manner [139]. In *srrAB* mutants, biofilm formation decreased over time and cell death levels increased under static aeration conditions compared with wild strains [140], indicating that the SrrAB is important for long-term biofilm stability and survival. This difference could be caused by different ways of hypoxia production, different growth media and different culture time. All these factors emphasize the importance of biofilm growth models [141]. 

The *S. aureus* exoprotein expression (SaeRS) TCS is a key regulator of toxin and exoprotein, which has critical roles in evasion of innate immunity and pathogenesis [142]. SaeRS TCS is composed of sensor kinase SaeS and response regulator SaeR along with two auxiliary proteins SaeP and SaeQ [143]. SaeP and SaeQ form a protein complex with SaeS, which activates the phosphatase activity of the SaeS and returns to the pre-activation state via a negative feedback mechanism [144]. SaeRS regulates the expression of genes associated with biofilm adhesion proteins such as *fnbA*, *fnbB,* and *fib* [109,145,146]. The accumulation of biofilm-related proteins enhances biofilm formation [147]. SaeRS mutation limits the biofilm formation of *S. aureus*. However, in *S. aureus* Newman, a point mutation in SaeS (SaeSP) leads to overexpression of SaeRS activity, preventing this strain from forming a robust biofilm, and exhibiting a SaeRS polymorphism [148]. In addition to SrrAB, SaeRS also regulates fermentation biofilm formation, decreased respiration caused an increasing activity in SaeRS, leading to increased expression of AtlA and FnbA as well as biofilm formation [149]. Recently, overexpression of ScrA (*S. aureus* clumping regulator A) was found causing an increase in bacterial aggregation [150]. Through proteomics and transcriptomics, strain overexpressing ScrA was found to cause cell aggregation and biofilm formation through activation of SaeRS, resulting in upregulation of multiple adhesins and downregulation of secreted proteases [150].

The cell death and lysis of *S. aureus* are regulated by LytSR TCS through a bacteriophage-like holin/antiholin system [151]. Holin is encoded by *cidABC* operon, CidA oligomerizes and forms pores in the cytoplasmic membrane, leading to membrane depolarization, activation of murine protein hydrolase activity, and cell lysis [151,152]. The CidA mutant exhibits decreased lysis, resulting in reduced eDNA content and impaired biofilm formation [153]. Antiholin inhibits the activity of CidA, encoded by *lrgAB* operon [81], which is an inhibitor of these processes [154], counteracts CidA activity by interfering with the ability of CidA to depolarize membranes and cause subsequent death and lysis [77]. The *lrgAB* operon, together with the *cidABC* operon, have been shown to be the regulators of cell death and lysis during biofilm development [152]. In *S. aureus*, LytSR positively regulates *lrgAB* operon and CidR enhances the expression of *cidABC* and *lrgAB* [155]. Agr and SarA, like LytSR, are positively regulating *lrgAB* expression. Mutations in the *agr* locus reduce *lrgAB* expression by approximately sixfold, whereas mutations in the *sarA* reduce *lrgAB* expression to undetectable levels [156]. *lytS* mutation results in more eDNA produced in ECM, leading to a thicker and more adherent biofilm [157]. Some studies have shown that the activity of the *lrgAB* promoter is mainly expressed in the tower structure of *S. aureus* biofilms [158], while a small part of the tower structure formed by *lytS* mutant strain still shows significant *lrgAB* expression. The results suggest that there is a LytS-independent pathway of LytR activation [159]. Since mutations in *srrAB* lead to increased cell death during biofilm development, it is reported that SrrAB inhibits cell death by directly suppressing the expression of the *cidABC* under conditions of glucose overload [160]. The cell death could be due to the effect of *cidABC* expression leading to increased reactive oxygen species (ROS) accumulation [160]. 

ArlRS TCS was originally identified as a regulator of autolysis and biofilm formation [161]. MgrA is a global transcriptional regulator downstream of ArlRS that forms a regulatory cascade with ArlRS [162]. ArlRS activates the expression of MgrA and regulates a variety of genes through MgrA, including seven CWPs and virulence [162]. Among the 15 mutants of non-essential TCSs, ArlRS TCS, SrrAB TCS, and Agr are required for biofilm formation, with ArlRS playing a major role in the process [163]. ArlRS enhances the expression of *icaADBC* by suppressing the expression of *icaR*, which activates PNAG production, and in *arl* mutants, the synthesis of PNAG is lost [163]. However, overexpression of MgrA could not restore PNAG expression in ArlRS mutants [164]. In contrast, MgrA can act as a negative regulator of *psm* expression, negatively regulating biofilm formation and dispersion by directly binding to the *psm* promoter region to repress transcription of the *psm* operon in cultures and biofilms [165]. These results suggest that ArlRS is a key TCS for biofilm formation.

### 4.5. The Second Messenger

Cyclic dinucleotides are highly versatile signaling molecules in prokaryotes involved in the control of various important biological processes [166]. These intracellular signal nucleotides coordinate diverse aspects of bacterial colony behavior, including motility, biofilm formation, and virulence gene expression [167]. The second messenger, cyclic diguanylic acid (c-di-GMP), is synthesized by two molecules of GTP by the diguanylate cyclases (DGCs) and degraded by the c-di-GMP phosphodiesterases (PDEs) [167], which is the main regulator of the conversion between free movement and biofilm of Gram-negative bacteria [168]. A high c-di-GMP level reduces the expression and/or activity of flagella and stimulates the synthesis of adhesins and biofilm-related extracellular polysaccharide [169], promoting biofilm formation [170]. In *S. aureus*, the second messenger, c-di-AMP, is synthesized by two molecules of ATP by the diadenylyl cyclase (DAC) enzyme DacA and degraded by the c-di-AMP phosphodiesterase GdpP-containing GGDEF domain [171]. Similar to c-di-GMP, in the *S. aureus* SEJ1 strain with *gdpP* mutation, the level of c-di-AMP and the amount of biofilm are significantly increased [171]. However, the *S. aureus* USA300 LAC strain could not form a robust biofilm in this condition [171], indicating that the effect of c-di-AMP on biofilm might be strain dependent. The *gdpP* mutation also leads to reduced eDNA levels, indicating that c-di-AMP is also involved in the release of eDNA [172]. In addition, The c-di-AMP also interacts with the purine biosynthesis pathway. In purine biosynthesis mutant methicillin-sensitive *S. aureus* (MRSA) strains, ADP, ATP, c-di-AMP, and eDNA levels were lower, and biofilm formation was less; vancomycin binding and its induced cleavage were increased [173]. In *S. aureus*, GdpS, a protein containing the GGDEF domain, inhibits early biofilm formation and is independent of autolysis by reducing *lrgAB* and *cidABC*-mediated release of eDNA [174]. These results reveal the influence of c-di-AMP on biofilms, which may play an important role in the persistence of *S. aureus* biofilm infection.

### 4.6. sRNAs

In addition to regulating transcriptional proteins, RNA molecules are now recognized as key players in gene regulation in all organisms [175]. Small RNAs (sRNAs) are usually non-coding and function at the level of transcription, translation, or RNA degradation [176], playing a key role in biofilm formation through base pairing with target mRNAs or interactions with regulatory proteins, resulting in both positive and negative regulatory mechanisms [177,178]. sRNA RsaA inhibits the synthesis of MgrA and enhances biofilm formation by binding to two regions of the mRNA of mgrA [179]. In addition, the synthesis of RsaA is controlled by SigB. Thus, RsaA functionally connects the global regulators SigB and MgrA [179]. The sRNA RsaF binds the hyaluronate lyase HsyA and serine protease-like protein SplD; the disruption of RsaF leads to a decreasing activity in HysA, which in turn increases biofilm formation [180]. sRNA RsaI (or RsaOG) depress the formation of biofilms at high glucose concentrations by binding to the 3′-UTR of *icaR*. Inhibition of IcaR synthesis by stabilization of mRNA recycles and/or by counteracts binding to activators of IcaR mRNA translation, but the exact molecular mechanism has not been determined. The unwinding of biofilm formation occurs by binding to the 3′-UTR of icaR. This result corroborates with previous reports of increased biofilm formation observed in hysA mutants [181]. The 5′-untranslated region of *sarA* contains two sRNAs, named teg49 and teg48, which are detectable in the P3-P1 and P1 regions of the *sarA* promoter, respectively [182]. sRNA teg58 plays an important role in regulating arginine biosynthesis and biofilm formation in *S. aureus*. teg58 inhibits arginine synthesis; the arginine biosynthesis gene (*argGH*) is upregulated in teg58 mutants. Biofilm formation is reduced in parental strains after supplementation with exogenous arginine or endogenous *argGH* [183]. However, teg49 does not affect biofilm formation. Biofilm-associated genes (such as *ica* locus) are not affected in the case of teg49 inactivation or overexpression [184]. Transcriptomic analysis suggests that teg49 may post-transcriptionally affect the SaeRS and LytRS TCS, but the exact mechanism is unclear [184]. The sRNA SprX is encoded in the pathogenicity island of the MRSA Newman strain. SprX1 is one of three copies of SprX. SprX1 interacts directly with mRNAs encoding ClfB and Hld. Cells overexpressing SprX1 exhibited increased intercellular aggregation and biofilm formation [185]. In addition, SprX may modulate its effect on biofilms by increasing the stability of RNAIII [185]. In general, the mechanism of sRNA in the regulatory pathway of biofilm formation is not very clear at present. Further exploration is still needed.

## 5. Conclusions and Future Perspectives

The presence of *S. aureus* and most bacteria in the form of a biofilm is a considerable challenge for the medical community. Biofilms are a survival strategy for bacteria, making them extremely difficult to treat due to their inherent immune response and antibiotic resistance. Therefore, it is necessary to further study the formation and regulation mechanism of *S. aureus* biofilm for research and development of anti-biofilm drugs that inhibit biofilm formation. The process of biofilm formation is complex and involves the co-expression of multiple genes. *S. aureus* relies on a broad network of regulatory systems that coordinate biofilm formation in a complementary or opposing way. At present, although progress has been made in the regulatory mechanism of the formation of *S. aureus* biofilm, researchers have not yet understood the synergy between different regulatory networks. Moreover, the regulation mechanism of biofilm is dynamic. For example, different growth times and different environments may have different regulatory genes. How to realize these signals thus needs further investigation. At the same time, little is known about whether the regulation mechanism of biofilm in vivo is the same as in vitro. Human organoid models may be one of the most exciting tools to understand the regulation mechanism of *S. aureus* biofilm. On the other hand, the experimental results of reference strains may not be similar to the results of clinical isolates. The results obtained from reference strains may have some differences from that of clinical isolates. Therefore, different clinical isolates can be used for further research to provide better clinical significance. In addition, in clinical infection, *S. aureus* can also form mixed species biofilm with other bacteria and/or fungi. It is also important to include mixed species biofilm in the study.

## Data Availability

Not applicable.
